# Case Report of a Solitary Brain Abscess due to *Nocardia veterana* in a Host Without Known Immunodeficiency

**DOI:** 10.1155/crdi/3980803

**Published:** 2026-05-17

**Authors:** Lauren Wells, Madeleine Purcell, Lo Tamburro, Joel V. Chua, Nazary Nebeluk, James B. Doub

**Affiliations:** ^1^ Department of Internal Medicine, University of Maryland School of Medicine, Baltimore, Maryland, USA, umaryland.edu; ^2^ Department of Pathology, University of Maryland School of Medicine, Baltimore, Maryland, USA, umaryland.edu; ^3^ Division of Clinical Care and Research, Institute of Human Virology, University of Maryland School of Medicine, Baltimore, Maryland, USA, umaryland.edu; ^4^ The Doub Laboratory of Translational Bacterial Research, University of Maryland School of Medicine, Baltimore, Maryland, USA, umaryland.edu

**Keywords:** brain abscess, *Nocardia veterana*, nocardiosis, trimethoprim-sulfamethoxazole

## Abstract

Nocardiosis is a rare bacterial infection caused by a wide array of species of the genus *Nocardia*. These microbes are found ubiquitously in soil and cause infection mainly when immunocompromised hosts inhale these bacteria or from direct inoculation through breaks in the skin. Once infection is established, *Nocardia* spp. have a predilection to spread to the central nervous system, causing brain abscesses. Here, we present a rare case of a solitary *Nocardia veterana* brain abscess in a patient without known immunodeficiency who was successfully treated with an 8‐week course of antibiotics. Overall, this case highlights an unusual bacterial pathogen in an atypical host successfully treated with a shorter course of antibiotics, an approach that may not be generalizable. It reinforces that opportunistic infections do occur in immunocompetent hosts, but clinical presentations may be obscured by limited symptoms and insidious courses.

## 1. Introduction

Nocardiosis is a rare aerobic actinomycetes bacterial infection caused by members of the *Nocardia* genus. *Nocardia* spp. are ubiquitous soil microbes that typically cause infections in immunocompromised hosts after bacteria are inhaled into the lungs or by direct inoculation through puncture wounds or breaks in the skin. Once the organism has entered the lung, it has a predilection to spread to the central nervous system (CNS), causing brain abscesses. Nocardiosis most frequently affects immunocompromised hosts due to impairments in their cell‐mediated immunity that fail to prevent disease dissemination. Treatment typically requires prolonged antibiotic therapy, with courses ranging from 6 months to 1 year. The mainstay of treatment is trimethoprim–sulfamethoxazole (TMP‐SMX), but other antibiotics such as carbapenems [1] and linezolid [2] have also been efficaciously used. Yet, the proper duration of antibiotic treatment is poorly defined, and for immunocompetent hosts, it is even less defined owing to the rarity of this disease in this population. Here, we present a rare case of a solitary *Nocardia veterana* brain abscess in a patient without known immunodeficiency that was successfully treated with a shorter course of antibiotics.

## 2. Case

A sixty‐eight‐year‐old female with a past medical history of hypertension presented to the emergency department (ED) with 5 days of progressively worsening headache. Initially, she thought her headache was secondary to changing her antihypertension medication to a generic form of amlodipine. However, the headache persisted, and she presented for evaluation. In the ED, she was evaluated and found to be afebrile with normal vital signs and an unremarkable neurological examination. She was prescribed ibuprofen and discharged with instructions to follow up with her primary care physician if symptoms persisted.

Her headache continued to worsen throughout the next week, causing her to again seek evaluation at her local ED. She underwent computed tomography (CT) of the head which did not show any acute intracranial abnormalities. Moreover, she continued to have normal vital signs and normal findings on neurological examination. As a result, she was discharged with instructions to follow up with her primary care physician and take ibuprofen as needed for her headache.

Unfortunately, her headache progressed over the next week, and she began to experience nausea and ataxia prompting her to go to the local ED for further evaluation. Symptoms at this point had been present for approximately 3 weeks in total. In the ED, complete blood count and comprehensive metabolic profile were normal. Hemoglobin A1c was measured at 6.2%, and HIV antibody screening was negative. Given the ataxia, a CT angiogram (CTA) of the brain was obtained which showed a right cerebellar rim‐enhancing lesion compressing the fourth ventricle. The patient was given dexamethasone, started on intravenous antibiotics (vancomycin, ceftriaxone, and metronidazole), and transferred to our tertiary care center for further management.

Upon questioning, the patient denied any recent travel, no outdoor hobbies, and no recent illnesses or sick contacts. Subsequent magnetic resonance imaging (MRI) was obtained which confirmed the CTA findings. MRI demonstrated a 3.3‐cm multilobulated, necrotic, rim‐enhancing mass and surrounding vasogenic edema involving the right cerebellar hemisphere (Figure 1). CT chest, abdomen and pelvis did not show any additional masses or abnormalities. As a result of these findings, neurosurgery planned and completed a stereotactic biopsy of the brain lesion. Beyond diagnostics, this procedure also included therapeutic drainage of purulent material to assist in source control. Infectious diseases consultation was obtained to assist with antimicrobial therapeutic management of possible brain abscess.

FIGURE 1MRI of brain. 3.3 cm multilobulated, necrotic, rim‐enhancing mass with corresponding restricted diffusion and associated moderate surrounding vasogenic edema involving the medial inferior aspect of the right cerebellar hemisphere and cerebellar vermis, consistent with abscess. (a) Axial view of T2 with sampling perfection with application optimized contrast; (b) coronal view of T1 fat‐saturated postcontrast sequence.

**Figure:**
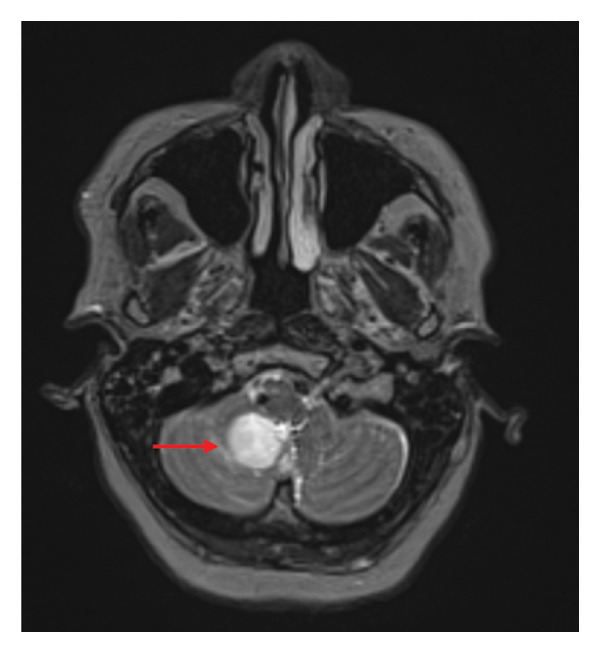
(a)

**Figure:**
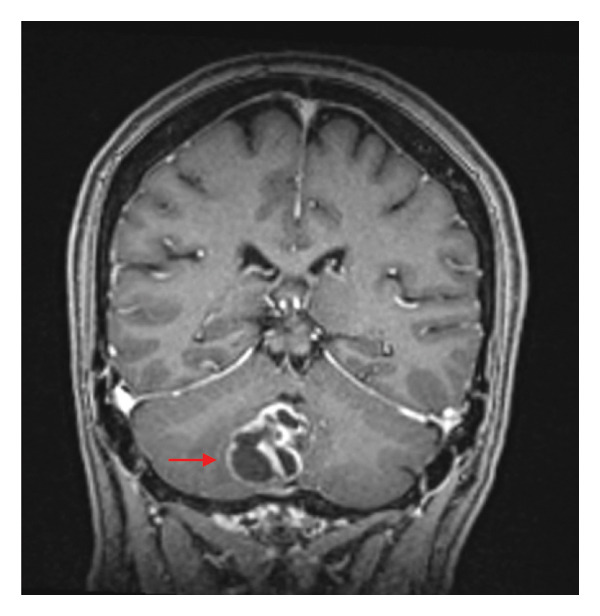
(b)

**FIGURE 2:**
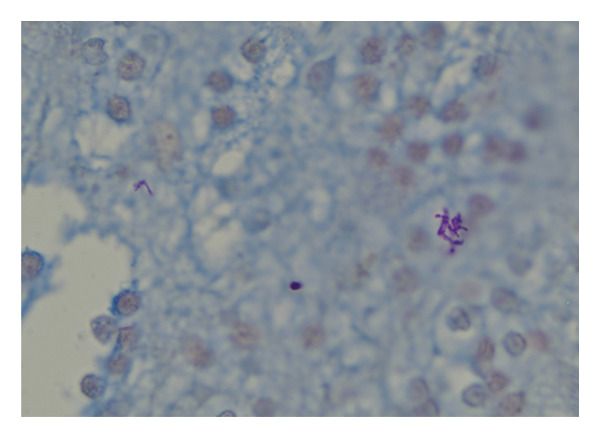
*Nocardia veterana* identified in cerebellar tissue biopsy. Light microscopic image of modified acid‐fast stain‐positive branched bacilli in cerebellar granular layer (Fite stain; ×1000 magnification). The associated AFB, PAS, and GMS special stains showed no specific staining of organisms. The tissue Gram stain showed nonspecific stain deposition that colocalized with the Fite‐positive organisms but did not show clear, specific staining of organisms. AFB, acid‐fast bacilli, PAS, periodic acid–schiff, GMS, Grocott’s methenamine silver.

The brain biopsy showed thick, yellow, purulent material with intraoperative frozen section revealing potential fungal elements. Therefore, the patient was started on amphotericin B in addition to the above‐mentioned antibacterial therapeutics. Gram stain from the abscess aspirate showed branched, beaded gram‐positive bacilli that were negative for acid‐fast staining (Kinyoun’s) and positive for modified acid‐fast staining, concerning for a *Nocardia* spp. These findings were concurrent with the organism staining profile seen in the biopsy tissue (Figure 2). Mass spectrometry of bacterial culture identified the organism as *Nocardia veterana*. Antimicrobial sensitivity testing was conducted via send‐out lab and performed via broth microdilution using custom‐made MIC panels. Sensitivity testing results are shown in Table 1. Consequently, antibiotics were changed to linezolid 600 mg oral every 12 h, TMP‐SMX 2 double strength tablets every 6 h (16.6 mg/kg/day) and imipenem 500 mg intravenous every 6 h. Even though there was no overt evidence of immunodeficiency, discussion with the patient was conducted about evaluation at the National Institutes of Health Immunodeficiency Clinic to ensure no obscure innate immune deficiency existed. The patient declined such evaluation.

**TABLE 1:** Antibiotic susceptibility testing of *Nocardia veterana* clinical isolate.

Antibiotic	MIC (μg/mL)	Interpretation
Amikacin	≤ 0.5	Susceptible
Amoxicillin/clavulanate	32/16	Resistant
Ceftriaxone	16	Intermediate
Ciprofloxacin	8	Resistant
Clarithromycin	0.06	Susceptible
Doxycycline	8	Resistant
Imipenem	1	Susceptible
Linezolid	1	Susceptible
Minocycline	4	Intermediate
Moxifloxacin	2	Intermediate
Tobramycin	128	Resistant
Trimethoprim/sulfamethoxazole	0.25/4.8	Susceptible

After the biopsy, her headache and ataxia improved. However, she developed nausea and vomiting secondary to linezolid, and this was discontinued 6 days after initiation. Prior to discharge, a peripherally inserted central catheter (PICC) was placed, and she was discharged to an acute rehab on dual therapy with IV imipenem via PICC line and oral TMP‐SMX (same dosing as above). Both imipenem and TMP‐SMX were continued for 8 weeks prior to infectious disease follow‐up. At the 8‐week follow‐up appointment, repeat MRI of the brain was completed which showed complete resolution of the abscess. Her symptoms had resolved as well. At this point, given radiologic and symptomatic resolution, both imipenem and TMP‐SMX were discontinued simultaneously at 8 weeks. There was no transition or oral step‐down regimen recommended after cessation of therapy. At a 1‐year follow‐up, the patient was not experiencing any residual symptoms or neurologic deficits to suggest disease recurrence.

## 3. Discussion

*Nocardia* spp. are found ubiquitously in soil and are gram‐positive aerobic bacilli that form characteristic beaded, branching filaments, similar in appearance to fungal hyphae at low power magnification. *Nocardia* spp. are typically weakly acid‐fast after traditional carbol‐fuchsin staining and positive on modified acid‐fast staining. Given the similarity to fungal hyphae, it is common for it to be confused for a fungal infection, as occurred in this case. Yet, after evaluation under high magnification with Gram stain, beaded gram‐positive rods were seen that were suggestive of *Nocardia* spp. Final diagnosis was made via mass spectrometry of bacterial culture; this method is often required to differentiate between *Nocardia* species given their phenotypic and genotypic similarities.

Until recently, knowledge surrounding the incidence of nocardiosis was based on estimated, historical survey data from the 1970s. Though no national reporting system for *Nocardia* spp. infections exists, more recent data provide updated insight into its incidence. One 2025 systematic review counted 9750 cases of *Nocardia* globally between the years 1950 and 2024 [3]. Approximately 60% of these cases occurred in immunocompromised hosts with the remaining 40% in immunocompetent hosts [4]. The main species responsible for most reported cases in immunocompromised hosts were *N. farcinica, N. abscessus* and N. *nova* complex (discussed in detail below). In this population, nocardiosis was more likely to present with disseminated disease [5]. It was previously thought that N. *asteroides*, since divided into 6 taxa, was a large contributor to a significant portion of these infections. More recently, *N. cyriacigeorgica* seems responsible for those previously attributed to the now outdated *N. asteroides* complex [6]. Comparatively, *N. brasiliensis* is the most likely species to infect immunocompetent hosts, often presenting as a cutaneous form of disease. In addition to immune status, *Nocardia* is known to be more common in males than in females, in a 3:1 ratio, and tends to appear in the fifth decade of life. There is no known racial predilection for infection [3].

In this case, a rare species, *Nocardia veterana*, was the causative agent. This species of Nocardia was first isolated in 2001 from bronchoalveolar lavage fluid at an Australian veteran’s hospital. It has more recently been included in the *N. nova* complex based on gene sequencing of isolates [6]. Since its discovery, *Nocardia veterana* has been a sparsely reported microbe [5, 7]. Similar to the genus at large, its incidence is poorly characterized. Of the 9750 cases discussed above, a recent 2021 case report found only 24 instances in which *Nocardia veterana* was identified and reported as the responsible bacteria. The majority of these were pulmonary infections in immunocompromised hosts, and only 3 were associated with abscesses in the bowel, soft tissue, and brain, respectively [8]. Of note, the brain abscess identified in that case was also due to *N. veterana* [9].

The usual method of *Nocardia* transmission is by inhalation of organisms suspended in dust. Thus, pulmonary nocardiosis is the most common type of infection. Another well‐known method of inoculation is by local traumatic introduction leading to cutaneous, lymphocutaneous and subcutaneous forms of nocardiosis. If not confined to the site of initial inoculation by the host immune system, *Nocardia* spp. disseminates via the bloodstream. The most common sites of dissemination are brain, kidneys, bone and muscle [4]. In our case, the patient’s likely source of exposure was inhalation. On review of history, she did not report any skin abrasions or infection, outdoor exposure or direct oral trauma. Given a likely pulmonary introduction, it is notable that the patient, in this case, did not have a respiratory prodrome nor any radiographic findings of pulmonary abnormalities. This suggests the possibility of impaired local pulmonary immune response to *Nocardia veterana* allowing for dissemination, despite the absence of a known systemic immunodeficiency.

The primary immune response to *Nocardia* exposure in immunocompetent hosts relies on an initial neutrophil and macrophage response followed by T‐lymphocytes. For this reason, there is a strong association between hosts that have T‐cell, neutrophil and macrophage deficiencies and increased risk for nocardiosis [10, 11]. The most common immunosuppressive conditions associated with nocardiosis are organ transplant, chemotherapy, acquired immune deficiency syndrome (AIDS) and chronic corticosteroid exposure [3, 12]. A prior case series of immunocompromised patients with CNS nocardiosis identified 84 patients, of whom half had been on long‐term corticosteroids, defined as a prednisone equivalent of > 10 mg/day for at least 3 months, and a quarter had a history of organ transplant [13]. Our patient did not have any of these immunosuppressing conditions. Furthermore, an initial workup including negative HIV testing, absence of diabetes, normal complete blood count, and no history of recurrent infections showed no evidence of an immunodeficiency. Further data such as CD4 counts or quantitative immunoglobulin studies were not obtained due to patient preference. She was recommended for comprehensive immune workup at the National Institute of Health Immunodeficiency Clinic however declined. For this reason, we refer to the patient as apparently immunocompetent due to limited, more detailed data.

Associated with host immune responses, *Nocardia* spp. have unique bacterial virulence factors that potentiate disease. The bacteria possess endogenous catalase and superoxide dismutase enzymes to inactivate the reactive oxygen species made by neutrophils. Further, a cord factor glycolipid in the cell wall is known to halt macrophage activation, fusion and phagocytosis [14]. This limited ability to activate macrophages and neutrophils at the site of infection thereby leads to robust localized infections that can then spread to adjacent tissues. Associated with this is the fact that *Nocardia* spp. possess surface receptors that have strong affinities to CNS capillary endothelial cells allowing for movement through the blood–brain barrier [15].

Once the blood–brain barrier is breached, CNS nocardiosis is associated with insidious, nonspecific neurologic symptoms that are gradual and persistent. This heterogeneity in presentation makes the syndrome difficult to recognize and can delay diagnosis, which increases the risks of morbidity and mortality. Symptoms can start broadly as a headache but progress to fever, vomiting, seizures or focal neurologic deficits due to mass effect from an abscess or granuloma [14]. The treatment of CNS nocardiosis is initially based on empiric antibacterial agents such as TMP‐SMX, linezolid and imipenem. In this case, treatment was initially broad with 3 antibacterial agents. However, targeted antimicrobial therapy should be initiated as soon as possible based on antimicrobial susceptibility testing (AST). This is because species identification influences drug selection and should be pursued as often as possible, as different species are resistant to different antibiotics. With 94 recognized species, 54 of them being clinically significant, the *Nocardia* genus has a myriad of diverse antimicrobial susceptibility profiles. A common theme among them is greater than 97% susceptibility to amikacin, linezolid and TMP‐SMX. Reported levels of TMP‐SMX resistance specifically have varied greatly; however, more recent studies have shown nearly 99% susceptibility across most *Nocardia* spp., notably *N. farcinica*, N. *brasilienses*, and *N. otitidiscaviarum* [16, 17].

[13, 18] With respect to *Nocardia veterana* specifically, a systematic analysis of antibiotic susceptibility profiles of seven case reports noted broad susceptibility to amikacin, ampicillin, imipenem and TMP‐SMX. Intermediate resistance to amoxicillin–clavulanic acid, ceftriaxone and minocycline was reported, with resistance to ciprofloxacin and occasionally vancomycin also reported [19]. Another case report notes similar findings, highlighting *Nocardia veterana* resistance to amoxicillin–clavulanic acid, ciprofloxacin and doxycycline with susceptibilities to imipenem, TMP‐SMX and linezolid [20]. In our case, the antimicrobial susceptibilities (Table 1) largely agree with prior reported data on *N. veterana*. However, none of the prior reported susceptibility data was collected from culture of a brain abscess in an apparently immunocompetent host, as in our case. Our patient underwent appropriate treatment selection based on species‐specific susceptibilities with TMP‐SMX, imipenem and linezolid.

Though data are minimal for *N. veterana* specifically, it is important to note its current inclusion in the *N. nova* complex which has wider known information on AST. Recent data reveal nearly 100% susceptibility to amikacin, imipenem, linezolid and TMP‐SMX with greater resistance to amoxicillin–clavulanic acid, ceftriaxone, clarithromycin and minocycline across the complex [16]. While these *N. nova* complex data largely agree with that of *N. veterana* above, it remains important to identify species as narrowly as possible to guide treatment decisions, especially when direct AST of a sample may not be feasible.

Of particular interest in this case is the relatively short, 8‐week course of antibiotic treatment. Current Infectious Disease Society of America clinical guidelines suggest 12 months of treatment for disseminated or CNS nocardiosis [21]. Treatment continues until both clinical and radiographic resolution. Shorter courses (< 3 months) are often reserved for cutaneous infection. Some research has been done on treatment duration in nocardiosis, but the topic is largely understudied. A recent multicenter study was performed comparing mortality in nocardiosis patients receiving standard (6–12 months) versus short (< 90 days) treatment duration. Short treatment duration was not associated with higher rates of relapse or increased mortality rates 1‐year postdiagnosis [22]. Patients in the short treatment group were more commonly less immunosuppressed (as in our case) but had a lower proportion of CNS involvement (unlike our case). In CNS involvement specifically, only 5.4% of patients were treated with a < 90 days course of antibiotics compared to 26.8% of patients who received > 180 days, making an eight‐week course (56 days) highly uncommon. Also relevant is the specific *Nocardia* species per case, with *N. farcinica* receiving prolonged treatment courses and being associated with poorer prognostic outcomes [22]. *N. veterana* was not identified in this study and may have had some impact on successful, shorter treatment duration in the presented case, though this is yet to be studied.

The conclusions above suggest that treatment duration should be assessed based on patient risk factors as well as clinical presentation and severity, allowing for safe use of shorter treatment courses in certain patients. While the patient in this case is apparently immunocompetent, CNS infection would generally be expected to require at least 6–12 months of treatment despite immune status. The presented 8 weeks of antibiotic therapy deviates significantly from guidelines and, while successful in this case, is drastically truncated. This treatment course should not be generalized and should be approached with caution in a dynamic patient‐centered decision‐making model where factors may make adherence to traditional therapy difficult.

## 4. Conclusion

This case highlights an uncommon etiology of a common medical complaint, headache. The patient was an unlikely host, and the organism, *N. veterana*, does not often cause profound disease. However, the case does emphasize that even apparently immunocompetent hosts can be susceptible to opportunistic infections, and they should be considered when patients have persistent, insidious symptoms. Brain biopsy was crucial to making a diagnosis in this case and reinforces the importance of elucidating microbial causes of infections and species identification as a means of tailoring antibiotic therapy. We further contribute to growing data surrounding safe antibiotic duration in CNS nocardiosis, though this case is highly unusual. Lastly, this case provides insight into the presentation and natural disease course of *N. veterana* and should spur more research into studying this genus and nocardiosis in general to determine optimal treatment regimens and their duration.

## Author Contributions

Lauren Wells, Madeleine Purcell, and Nazary Nebeluk contributed to chart review and manuscript preparation; Lo Tamburro and James B. Doub contributed to figure preparation; and Nazary Nebeluk, Joel V. Chua, and James B. Doub contributed to manuscript editing.

## Funding

None of the authors received specific funding for this project.

## Consent

Informed consent was obtained from the patient for publication of this case report and accompanying clinical images.

## Conflicts of Interest

The authors declare no conflicts of interest.

## Data Availability

All data pertinent to this case report are contained within the manuscript.

## References

[1] Dhakal D. , Abdulla M. C. , Qaboos Hospital S. , Yishan Lu O. , Shen Y. , and Ye Y. , Nocardiosis: A two-center Analysis of Clinical Characteristics.

[2] Davidson N. , Grigg M. J. , McGuinness S. L. , Baird R. J. , and Anstey N. M. , Safety and Outcomes of Linezolid Use for Nocardiosis, Open Forum Infectious Diseases. (2020) 7, no. 4, 10.1093/ofid/ofaa090. PMC7112726 32258209

[3] Du B. , Song Z. , and Ren Z. , The Global Epidemiology, Risk Factors, and Mortality Prediction of Nocardiosis: an Easily Missed Opportunistic Infection, Scientific Reports. (2025) 15, no. 1, 10.1038/s41598-025-26244-1. PMC12657858 41298615

[4] Cdc , Clinical Overview of Nocardiosis.

[5] Pottumarthy S. , Limaye A. P. , Prentice J. L. , Houze Y. B. , Swanzy S. R. , and Cookson B. T. , Nocardia veterana, a New Emerging Pathogen, Journal of Clinical Microbiology. (2003) 41, no. 4, 1705–1709, 10.1128/JCM.41.4.1705-1709.2003, 2-s2.0-0037387126. 12682164 PMC153934

[6] Conville P. S. , Brown-Elliott B. A. , Smith T. , and Zelazny A. M. , The Complexities of Nocardia Taxonomy and Identification, 2017. 10.1128/JCM.01419-17 PMC5744224 29118169

[7] Gürtler V. , Smith R. , Mayall B. C. , Pötter-Reinemann G. , Stackebrandt E. , and Kroppenstedt R. M. , Nocardia veterana Sp. Nov., Isolated from Human Bronchial Lavage, International Journal of Systematic and Evolutionary Microbiology. (2001) 51, no. 51, 933–936, 10.1099/00207713-51-3-933, 2-s2.0-0034986426. 11411717

[8] Radcliffe C. , Peaper D. , and Grant M. , Nocardia veterana Infections: Case Report and Systematic Review, New Microbes New Infections. (2020) 39, 10.1016/j.nmni.2020.100833. PMC7797559 33456780

[9] Arends J. E. , Stemerding A. M. , Vorst S. P. , De Neeling A. J. , and Weersink A. J. L. , First Report of a Brain Abscess Caused by Nocardia veterana, Journal of Clinical Microbiology. (2011) 49, no. 12, 4364–4365, 10.1128/JCM.01062-11, 2-s2.0-82455185071. 21998437 PMC3232992

[10] Uttamchandani R. B. , Daikos G. L. , Reyes R. R. et al., Nocardiosis in 30 Patients with Advanced Human Immunodeficiency Virus Infection: Clinical Features and Outcome, 1994, http://cid.oxfordjournals.org/. 10.1093/clinids/18.3.348 8011814

[11] Lafont E. , Marciano B. E. , Mahlaoui N. et al., Nocardiosis Associated with Primary Immunodeficiencies (Nocar-DIP): an International Retrospective Study and Literature Review, Journal of Clinical Immunology. (2020) 40, no. 8, 1144–1155, 10.1007/s10875-020-00866-8. 32920680

[12] Tomás R. M. , Menéndez Villanueva R. , Reyes Calzada S. et al., Pulmonary Nocardiosis: Risk Factors and Outcomes, Respirology. (2007) 12, no. 3, 394–400, 10.1111/j.1400-1843.2007.01078.x. 17539844

[13] Anagnostou T. , Arvanitis M. , Kourkoumpetis T. K. , Desalermos A. , Carneiro H. A. , and Mylonakis E. , Nocardiosis of the Central Nervous System: Experience from a General Hospital and Review of 84 Cases from the Literature, Medicine (United States). (2014) 93, no. 1, 19–32, 10.1097/MD.0000000000000012, 2-s2.0-84892986957. PMC4616325 24378740

[14] Beaman B. L. and Beaman L. , Nocardia Species: Host-Parasite Relationships, 1994, https://journals.asm.org/journal/cmr. 10.1128/cmr.7.2.213 PMC358319 8055469

[15] Ji X. , Han L. , Zhang W. et al., Molecular, Cellular and Neurological Consequences of Infection by the Neglected Human Pathogen Nocardia, BMC Biology. (2022) 20, no. 1, 10.1186/s12915-022-01452-7. PMC9647956 36352407

[16] Hamdi A. M. , Fida M. , Deml S. M. , Abu Saleh O. M. , and Wengenack N. L. , Retrospective Analysis of Antimicrobial Susceptibility Profiles of *Nocardia* Species from a Tertiary Hospital and Reference Laboratory, 2011 to 2017, Antimicrobial Agents and Chemotherapy. (2020) 64, no. 3, 10.1128/AAC.01868-19. PMC7038297 31818815

[17] Zhao P. , Zhang X. , Du P. , Li G. , Li L. , and Li Z. , Susceptibility Profiles of Nocardia spp. to Antimicrobial and Antituberculotic Agents Detected by a Microplate Alamar Blue Assay, Scientific Reports. (2017) 7, no. 1, 10.1038/srep43660, 2-s2.0-85014500531. PMC5333629 28252662

[18] Hershko Y. , Levytskyi K. , Rannon E. et al., Phenotypic and Genotypic Analysis of Antimicrobial Resistance in Nocardia Species, Journal of Antimicrobial Chemotherapy. (2023) 78, no. 9, 2306–2314, 10.1093/jac/dkad236. 37527397

[19] Ansari S. R. , Han X. Y. , O’Brien S. , and Safdar A. , Nocardia veterana Bloodstream Infection in a Patient with Cancer and a Summary of Reported Cases, International Journal of Infectious Diseases. (2006) 10, no. 6, 483–486, 10.1016/j.ijid.2006.03.005, 2-s2.0-33750318670. 16876454

[20] Poisnel E. , Roseau J. B. , Landais C. , Rodriguez-Nava V. , Bussy E. , and Gaillard T. , Nocardia Veterana: Disseminated Infection with Urinary Tract Infection, Brazilian Journal of Infectious Diseases. (2015) 19, no. 2, 216–219, 10.1016/j.bjid.2014.11.003, 2-s2.0-84925847904. PMC9425234 25636185

[21] Yetmar Z. A. , Marty P. K. , Clement J. , Miranda C. , Wengenack N. L. , and Beam E. , Executive Summary of State-of-the-Art Review: Modern Approach to Nocardiosis—Diagnosis, Management, and Uncertainties, Clinical Infectious Diseases. (2025) 80, no. 4, 701–702, 10.1093/cid/ciae644. 40305687

[22] Attias N. H. , Schlaeffer-Yosef T. , Zahavi I. et al., Shorter vs. standard-duration Antibiotic Therapy for Nocardiosis: a multi-center Retrospective Cohort Study, Infection. (2025) 53, no. 3, 1115–1127, 10.1007/s15010-024-02445-0. 39589427 PMC12137364

